# Tension pneumothorax, is it a really life-threatening condition?

**DOI:** 10.1186/1749-8090-8-197

**Published:** 2013-10-15

**Authors:** Jeong Seob Yoon, Si Young Choi, Jong Hui Suh, Jin Yong Jeong, Bae Young Lee, Yong Gue Park, Chi Kyung Kim, Chan Beom Park

**Affiliations:** 1 Department of Thoracic and Cardiovascular Surgery, Incheon St. Mary’s Hospital, The Catholic University of Korea, 665-8, Bupyeong-dong, Bupyeong-gu, Incheon 403-720, Republic of Korea; 2 Department of Thoracic and Cardiovascular Surgery, Uijeongbu St. Mary’s Hospital, The Catholic University of Korea, Uijeongbusi, Korea; 3 Department of Radiology, St. Paul’s Hospital, The Catholic University of Korea, Seoul, Korea; 4 Department of Biostatistics, College of Medicine, The Catholic University of Korea, Seoul, Korea; 5 Department of Thoracic and Cardiovascular Surgery, St. Paul’s Hospital, The Catholic University of Korea, Seoul, Korea

**Keywords:** Tension pneumothorax, Bullae, Thoracostomy

## Abstract

**Background:**

Tension pneumothorax is a life-threatening occurrence that is infrequently the consequence of spontaneous pneumothorax. The aim of this study was to identify the risk factors for the development of tension pneumothorax and its effect on clinical outcomes.

**Methods:**

We reviewed patients who were admitted with spontaneous pneumothorax between August 1, 2003 and December 31, 2011. Electronic medical records and the radiological findings were reviewed with chest x-ray and high-resolution computed tomography scans that were retrieved from the Picture Archiving Communication System.

**Results:**

Out of the 370 patients included in this study, tension pneumothorax developed in 60 (16.2%). The bullae were larger in patients with tension pneumothorax than in those without (23.8 ± 16.2 mm vs 16.1 ± 19.1 mm; *P* = 0.007). In addition, the incidence of tension pneumothorax increased with the lung bulla size. Fibrotic adhesion was more prevalent in the tension pneumothorax group than in that without (*P* = 0.000). The bullae were large in patients with fibrotic adhesion than in those without adhesion (35.0 ± 22.3 mm vs 10.4 ± 11.5 mm; *P* = 0.000). On multivariate analysis, the size of bullae (odds ratio (OR) = 1.03, *P* = 0.001) and fibrotic adhesion (OR = 10.76, *P* = 0.000) were risk factors of tension pneumothorax. Hospital mortality was 3.3% in the tension pneumothorax group and it was not significantly different from those patients without tension pneunothorax (*P* = 0.252).

**Conclusions:**

Tension pneumothorax is not uncommon, but clinically fatal tension pneumothorax is extremely rare. The size of the lung bullae and fibrotic adhesion contributes to the development of tension pneumothorax.

## Background

Spontaneous pneumothorax is regarded as a common and benign clinical entity, however, it can be life-threatening if it progress to tension pneumothorax. While tension pneumothorax can develop abruptly, cardiovascular compromise progress more gradually due to the existence of a compensatory mechanism. However, sudden deterioration may occur once a critical point is passed, and so early recognition and prompt intervention before hemodyanamic deterioration is important [1].

Tension pneumothorax is characterized by progressive tachycardia, respiratory distress, sweating, hypotension and pallor resulting from hypoxemia, mediastinal shift and reduced venous return. Fatal cardiopulmonary collapse can occur if it remains untreated. However, large-series study of clinical outcomes in patient with tension pneumothorax are lacking. The results of several studies have suggested that tension pneumothorax is an uncommon medical emergency that requires immediate decompression [2-4]. However, the incidence, pathophysiology, and hospital outcomes of tension pneumothorax have yet to be established.

The aim of this study is to determine the overall incidence of tension pneumothorax in patients with spontaneous pneumothorax, the risk factors for the development of tension pneumothorax and its impact on the clinical outcome.

## Methods

After receiving approval (no. PC12RISI0058) from the institutional review board of St. Paul’s Hospital, patients who admitted with diagnosis of pneumothorax between August 2003 and December 2011 were identified. The board waived informed consent from the patients. Patients with defined traumatic or iatrogenic pneumothorax were excluded, as were those for whom an initial x-ray could not be found including one patient who was transferred from another hospital with a tube thoracostomy. After exclusion, 370 episodes in 302 patients were included in this study.

Data were collected by a retrospective review of electronic medical records, including the operative notes. Radiologic findings of pneumothorax were assessed with chest x-ray and high-resolution computed tomography (HRCT) scans, which were retrieved from the Picture Archiving Communication System (PACS) of the hospital. The HRCT scan of 309 (83.5%) patients were checked. Chest x-RAY and CT scans that were obtained in the same center were reviewed by a thoracic radiologist (B.Y.L.) and a thoracic surgeon (C.B.P.).

Tension pneumothorax was defined as follows: (1) hemodynamic compromise accompanied by tachycardia, tachypnea, sweating, hypotension and pallor, (2) hemodynamic improvement and release of gas after tube thoracostomy, (3) mediastinal shifting including trachea deviation toward the opposite site of the pneumothorax, a pushed cardiac silhouette, crossing over the spine of air density, compression, shifting of left cardiac border, and flattening of the diaphragmatic contour, (4) return of the shifted mediastinal structure after tube thoracostomy.

The area of pneumothorax (Area_pneumothorax_) and involved hemithorax (Area_hemithorax_) was measured on a chest radiograph using a picture archiving communication system (PACS; Marosis m-view, Infinitt, Korea) with an automated region of interest (ROI) calculator by a thoracic radiologist (B.Y.L.) and a thoracic surgeon (C.B.P.). The pneumothorax size was determined using the following formula:

$$\mathrm{Size}\;\mathrm{of}\;\mathrm{pneumothorax}\left(\%\right)=\left({\mathrm{Area}}_{\mathrm{pneumothorax}}/{\mathrm{Area}}_{\mathrm{hemithorax}}\right)100$$

In the following, “total events” refers to the sum of the frequency of ipsilateral and contralateral occurrences of pneumothorax, “ipsilateral recurrence” indicates the number of occurrences of an ipsilateral pneumothorax, and “fibrotic adhesion” is defined as the radiologic findings of diffuse joining of the parietal pleura and the visceral pleura or the presence of adhesive band. Reexpansion pulmonary edema (REPE) was diagnosed radiologically as the following criterias : (1) ipsilateral ground-glass opacities, (2) interlobular septal thickening or intralobular interstitial thickening, (3) consolidation, and (4) atelectasis. The size of the bullae was defined by the longest diameter of the largest bullae.

Statistical analyses were performed using SPSS (version 17.0, SPSS, Chicago, Illinois, USA). Data are presented as either mean ± standard deviation or as frequences and percentages, as appropriate. Continuous variables were compared using the independent two-sample *t* test or Mann–Whitney *U* test, and categorical variables were compared using the chi square or Fisher’s exact test, as appropriate. To identify independent risk factors for tenson pneumothorax, stepwise logistic regression analysis was performed. As the mean size of the bullae was closely associated with the presence of fibrotic adhesion, we adopted two models of regression analysis (Table 1). The level of statistical significance was set at *P* < 0.05.

**Table 1:** Multivariate analysis for the development of tension pneumothorax

		**Variable**	**Odds ratio**	**95% Confidence interval**	***P *** **value**
Model1		Size of largest bullae	1.03	1.01 – 1.04	0.001
		Size of pneumothorax	1.07	1.05 – 1.09	<0.001
Model2		Fibrotic adhesion	10.76	4.69 – 24.7	<0.001
		Size of pneumothorax	1.09	1.06 – 1.11	<0.001

Model1: size of largest bullae and size of pneumothorax were entered on analysis.

Model2: fibrotic adhesion and size of pneumothorax were entered on analysis.

## Results

Our final cohort comprised 370 patients; the demographic characteristics are described in Table 2. The age of the patients was 40.1 ± 21.8 years, ranging from 15 to 82 years, and 325 (87.8%) of them were male. The prevalence of tension pneumothorax was 16.2%. The prevalences of first-attack and recurrent-attack pneumothorax were 70.3% (*n* = 260) and 29.7% (*n* = 110), respectively.

**Table 2:** Patient demographics

**Variable**	**With tension**		**Without tension**		***P *** **value**
	**Pneumothorax**		**Pneumothorax**		
	**(*n*** **= 60)**		**(*n*** **= 310)**		
Age (years)	52.1 ± 21.2		37.8 ± 21.2		<0.001
Sex (male)	52	(86.7%)	273	(88.1%)	0.762
Diagnosis					<0.001
Primary	24	(40.0%)	204	65.8%)	
Secondary	36	(60.0%)	106	(34.2%)	
Diabetes mellitus	3	(5.0%)	13	(4.2%)	0.731
Hypertension	14	(23.3%)	27	(8.7%)	0.001
Tuberculosis	24	(40.0%)	77	(24.8%)	0.016
COPD	24	(40.0%)	67	(21.6%)	0.002
Smoking	32	(55.2%)	117	(42.5%)	0.079

COPD, chronic obstructive pulmonary disease.

The patients with tension pneumothorax were older than those without (52.1 ± 21.2 years vs 37.8 ± 21.2 years, *P* < 0.001). The prevalence of secondary pneumothorax differed according to the presence (60.0%) or absence (34.2%) of tension pneumothorax (*P* < 0.000). Among patients with tension pneumothorax, the prevalence of history of hypertension (23.3% vs 8.7%, *P* = 0.001), tuberculosis (40.0% vs 24.8%, *P* = 0.016), and chronic obstructive pulmonary disease (COPD; 40.0% vs 21.6%, *P* = 0.002) was higher than in patients without tension pneumothorax (Table 2).

**Figure 1:**
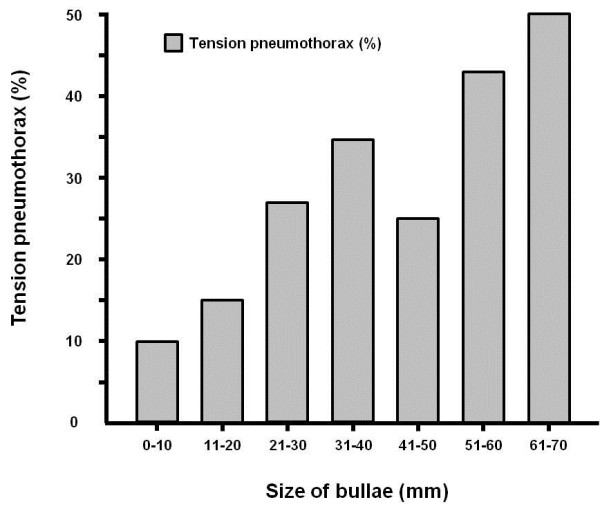
The incidence of tension pneumothorax increases with the size of the largest bullae.

The lung bullae were larger in patients with tension pneumothorax than in those without (23.8 ± 16.2 mm vs 16.1 ± 19.1 mm, *P* = 0.007) (Table 3). The incidence of tension pneumothorax increased with the bulla size (Figure 1).

**Table 3:** Characteristics of pneumothorax and radiologic findings

**Variable**	**With tension**	**Without tension**	***P *** **value**
	**Pneumothorax**	**Pneumothorax**	
	**(*n*** **= 60)**	**(*n*** **= 310)**	
Total events	1.48 ± 0.93	1.46 ± 0.88	0.881
Ipsilateral recurrence	1.30 ± 0.53	1.27 ± 0.60	0.757
Site (right : left)	26:34	162:148	0.206
Reexpansion pulmonary	22 (36.7%)	25 (11.3%)	<0.001
Edema			
Fibrotic adhesion	34 (56.7%)	84 (27.3%)	<0.001
Size of pneumothorax (%)	52.6 ± 17.4	29.0 ± 18.1	<0.001
Size of bullae (mm)	23.8 ± 16.2	16.1 ± 19.1	0.007

**Figure 2:**
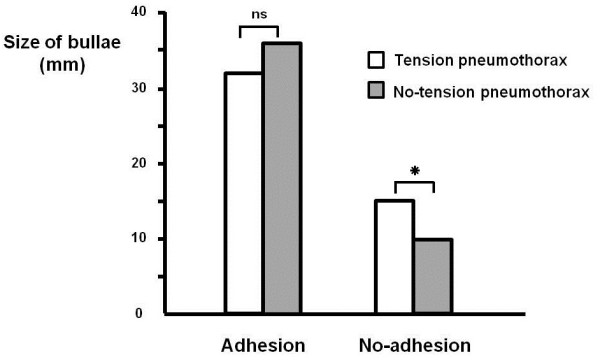
**Comparison of bulla size relative to the presence of tension pneumothorax.** Among patients without fibrotic adhesion, the bullae were larger in the tension pneumothorax group than in those without tension pneumothorax (15.2 ± 12.7 mm vs 9.8 ± 11.2 mm, *P* = 0.025). However, in cases where fibrotic adhesion was present, the size of the bullae did not differ significantly between the groups (32.0 ± 15.1 mm vs 36.3 ± 24.8 mm; *P* = 0.323). ns, not statistically significant; * *P* 0.05.

Fibrotic adhesion was more prevalent in the tension pneumothorax group than in the group without tension pneumothorax group (56.7% vs 27.3%, *P* < 0.001). The bullae were larger in patients with fibrotic adhesion than in those without (35.0 ± 22.3 mm vs 10.4 ± 11.5 mm, *P* = <0.001). However, among those patients who exhibited adhesion, bulla size did not differ between patients with tension pneumothorax and those without (32.0 ± 15.1 mm vs 36.3 ± 24.8 mm, *P* = 0.323) (Figure 2).

The predictors for the tension pneumothorax were identified by multivariate analysis. The size of largest bullae (odds ratio (OR), 1.03; *P* = 0.001), the presence of fibrotic adhesion (OR,10.76; *P* < 0.001) and the size of pneumothorax were independent risk factors for tension pneumothorax (Table 1).

Among patients who underwent operation, the preoperative and the postoperative hospital stays were longer in the tension pneumothorax group than in those without; however, the duration of chest tube drainage was not significant (*P* = 0.209) (Table 4). Hospital mortality was higher in tension pneumothorax group, but statistically insignificant (3.3% vs 1.3%, *P* = 0.252).

**Table 4:** Treatment and hospital outcomes

**Variable**	**With tension pneumothorax**		**Without tension pneumothorax**	***P *** **value**
	**(*n*** **= 60)**		**(*n*** **= 310)**	
Treatment method				0.020
O_2_ supplement		0 (0%)	23 (7.8%)	
CTD	29 (48.3%)		104 (33.8%)	
Operation	31 (51.7%)		181 (58.8%)	
Operation method				
VATS	29 (67.4%)		119 (65.7%)	
Open thoracotomy	14 (32.6%)		62 (34.3%)	
Preoperative hospital stay				
		6.3 ± 5.1	2.9 ± 2.9	0.001
In operation group (days)				
Postoperative hospital stay			5.2 ± 4.5	0.015
		8.2 ± 6.0		
In operation group (days)				
CTD removal (days)		5.3 ± 7.4	3.9 ± 5.5	0.099
Operation		3.5 ± 3.0	2.8 ± 3.0	0.209
No operation		7.5 ± 10.2	6.0 ± 7.9	0.425
Hospital stay (days)		11.6 ± 9.2	8.0 ± 7.5	0.007
Operation		13.6 ± 10.1	8.1 ± 6.2	0.006
No operation		9.4 ± 7.8	8.1 ± 9.1	0.459
Hospital mortality	2 (3.3%)		4 (1.3%)	0.252

*CTD*, chest tube drainage; *VATS*, video-assisted thoracoscopic surgery.

## Discussion

The findings of the present study demonstrate that the bullae were larger in cases of tension pneumothorax than in those without tension pneumothorax, and the prevalence of tension pneumothorax increased with the bulla size. The seconding finding is that prevalence of tension pneumothorax was not uncommon and fatal outcome was very unusual.

The underlying mechanism of tension pneumothorax is known as check valve system [2,4]. When a one-way valve is created between the lung and the pleura, air accumulates in the pleural cavity during the respiratory cycle and the consequent increase in intrapleural pressure interferes with the effective expansion of the lung on the side of pneumothorax. Increasing pressure in the pleural cavity aggravates the ipsilateral lung collapse and pushes the heart and mediastinal structures toward the other side of the chest. The vena cava and right heart become compressed, venous return is compromised, and diastolic filling and cardiac output are decreased. Significant shunting with ventilation-perfusion mismatching leads to hypoxemia, acidosis, and shock.

The relationship between the tension pneumothorax and bulla size has not been reported previously. According to Laplace’s law, wall tension increases with the radius. The tension would be higher on the walls of large bullae than on smaller bullae, so making the bullae walls thinner could predispose rupture more easily. Rupture of large bullae may result in a higher flow and higher pressure in the pleural space than rupture of small bullae. Progressive accumulation of high-flow and high-pressure in the limited pleural space leads to the compression of the mediastinal structure.

In our study, patients who had tension pneumothorax had a higher prevalence of fibrotic adhesion on chest x-ray. This finding is associated with higher incidence of secondary pneumothorax, tuberculosis, and COPD. Fibrotic adhesion is commonly observed in these clinical conditions, together with large bullae. The finding that the bullae was larger in patients with fibrotic adhesion than in patients without (35.0 ± 22.3 mm vs 10.4 ± 11.5 mm, *P* = 0.000) could be associated with the higher incidence of tension pneumothorax in patients with fibrotic adhesion. A subgroup analysis of patients with fibrotic adhesion disclosed that bullae size was not associated with the development of tension pneumothorax (32.0 ± 15.1 mm vs 36.3 ± 24.8 mm, *P* = 0.323). However, among patients without adhesion, the bullae were significantly larger in the tension pneumothorax group than in those without tension pneumothorax (15.2 ± 12.7 mm vs 9.8 ± 11.2 mm; *P* = 0.025). This finding indicates that the presence of fibrotic adhesion is another important risk factor for the development of tension pneumothorax.

The findings of a higher prevalence of hypertension in tension pneumothorax is interesting. Cardiovascular disease is more frequent in COPD, and COPD is an independent risk factor for cardiovascular disease [5,6]. In our study, tension pneumothorax was more common in cases with COPD. Hypertension combined with COPD might potentiate the risk of the development of tension pneumothorax. We did not investigate factors related to cardiovascular disease other than hypertension, further study regarding the relationship between tension pneumothorax and cardiovascular disease is needed.

It has been known that the incidence of tension pneumothorax is rare. In 1965, Mills and Baisch reported 14 cases of tension pneumothorax (3.5%) among 400 cases of spontaneous pneumothorax [7]. Since then, the incidence of tension pneumothorax has been variably reported as 0.5%–35.9% [7-13]. However, none of these studies were conducted specifically to examine tension pneumothorax. Our series of 60 cases of tension pneumothorax in a cohort of 370 patients (i.e., 16.2%) is the largest study population examined to date in this field, which means that the incidence of tension pneumothorax reported here reflects the situation more accurately than those reported previously.

Among patients who underwent surgery, the preoperative and postoperative hospital stay was longer in the tension pneumothorax group than in those without tension pneumothorax. However, the duration of chest tube drainage did not differ between these two groups (*P* = 0.209). These findings suggest that tension pneumothorax does not affect the operative outcome (duration of chest tube drainage), but it does influence the hospital stay. Its influence on hospital stay is attributable to the accompanying comorbidities including age, hypertension, COPD, and REPE.

There were six hospital mortalities in our study population, two of which were included in the tension pneumothorax group (3.3%). One-65-year old male patient was dead on arrival at the hospital. Despite immediate tube thoracostomy, he passed away with adult respiratory distress syndrome and hypoxic brain damage. Another 73-year-old male patient had terminal lung cancer combined with pneumonia. He died of sepsis 2 days after hospital admission. These findings corroborate the accepted knowledge that fatal tension pneumothorx is extremely rare, and suggest that the poor prognosis of tension pneumothorax is attributable to associated comorbidities rather than the tension pneumothorax itself.

Our study was subject to limitation. We adopted tension pneumothorax as a radiologic diagnosis whether it included clinical symptoms and signs of tension pneumothorax or not. Diagnosing tension pneumothorax is difficult due to the absence of classic signs [3,4]. Chest signs have been noted but they are poorly correlated with the condition and diagnosis is often missed before the chest x-ray. Chest radiolography confirms a tension pneumothorax even in patients without clinical symptoms or unstable vital sign [3]. However, radiologically detected tension pneumothorax without significant hemodynamic instability has the same pathophysiology. Even if the patient is young, with good cardiopulmonary reserve and clinically stable, the patient is at risk of sudden deterioration and possible cardiac arrest. Progressive accumulation of air in the pleural space and the decrease in oxygen saturation does not appear to influence the mean arterial pressure and heart rate until an involvement by pneumothorax of approximately 47% of the total lung capacity occurrs [1]. After this point, hemodynamic deteorioration (or “tension physiology”) appears as a late finding. Even a radiologic tension pneumothorax without hemodynamic instability still has the potential to progress to cardiorespiratory collapse. Therefore, a high suspicion of tension pneumothorax and immediate treatment after rapid radiologic confirmation is important even if the patient is hemodymaically stable.

## Conclusion

In conclusion, although tension pneumothorax is not uncommon, clinically fatal tension pneumothorax is extremely rare. The risk of tension pneumothorax is associated with the size of the largest bullae and the presence of fibrotic adhesion. Although tension pneumothorax can present without hemodynamic instability, it requires immediate decompression because significant hemodynamic deterioration can occur as a late manifestation.

## Abbreviations

HRCT: High resolution computed tomography; PACS: Picture archiving communication system; ROI: Region of interest; REPE: Reexpansion pulmonary edema; COPD: and chronic obstructive pulmonary disease; OR: Odds ratio.

## Competing interests

The authors declare that they have no competing interests.

## Authors’ contributions

JSY contributed to writing and critical revision of the manuscript. SYC contributed to writing the manuscript. JHS contributed to statistical analysis. JYJ contributed to collecting data. BYL contributed to analysis of radiologic findings. YGP contributed to statistical analysis. CKK contributed to interpretation of data. CBP was responsible for the integrity of the work and edited manuscript. All authors read and approved the final manuscript.
